
JOURNAL INFORMATION
==============================
NLM Title Abbreviation: ScientificWorldJournal
Journal ID: ScientificWorldJournal
NLM Journal ID: 101131163
Journal ID: TSWJ

The Scientific World Journal

ISSN: 2356-6140
EISSN: 1537-744X
Publisher: Wiley

ARTICLE INFORMATION
==============================
PMCID: PMC6084103
PMID: 30147568
DOI: 10.1100/tsw.2001.195
Article ID: 457627
Article version: 1
Subjects: Short Report

Stress-Activated Protein Kinases (SAPKs) in IGF-I Mediated Cell Survival

Krause Darren
Lyons Anthony
Walsh Patrick
O’Connor Rosemary
Cell Biology Laboratory, Biochemistry Department, Biosciences Research Institute, National University of Ireland, Cork, Ireland
Publication date: 2001
Electronic publication date: 2001 Dec 8
Volume: 1
First page: 94
Last page: 94
Copyright: Copyright © 2001 Darren Krause et al.
Copyright year: 2001
License: This is an open access article distributed under the Creative Commons Attribution License, which permits unrestricted use, distribution, and reproduction in any medium, provided the original work is properly cited.
License URL: https://creativecommons.org/licenses/by/3.0/

==============================
Miami *Nature Biotechnology* Short Reports
*TheScientificWorld* (2001) 1 (S3), 94SR
ISSN 1532-2246; DOI 10.1100/tsw.2001.195

### STRESS-ACTIVATED PROTEIN KINASES (SAPKS) IN IGF-I MEDIATED CELL SURVIVAL

Darren Krause, Anthony Lyons, Patrick Walsh, and Rosemary O'Connor

Cell Biology Laboratory, Biochemistry Department, Biosciences Research Institute, National University of Ireland, Cork, Ireland

**INTRODUCTION.** The IGF-I receptor is a potent mediator of cell survival in response to diverse death stimuli in different tissues. Several signalling pathways can be activated by the IGF-IR including the PI-3 kinase/AKT pathways leading to cell death regulators. However, there is also evidence for IGF-I-mediated survival signalling that is not dependent on PI-3 kinase and AKT activation. Here, we investigated the stress activated protein kinases (SAPKs) including Jun N-terminal kinase (JNK) and p38 as candidate mediators of PI-3 kinase independent survival signalling.

**RESULTS.** Initial experiments demonstrated that IGF-I elicits transient phosphorylation of JNK and c-Jun in FL5.12/IGF-IR cells (1) even in the presence of the PI-3 kinase inhibitor LY294002. To determine if JNK contributes to IGF-I-mediated survival signalling we used the quinone reductase inhibitor dicoumarol, which inhibits JNK activation (2). Dicoumarol suppressed IGF-I-induced phosphorylation of c-Jun and caused a dose-responsive abrogation of IGF-I-mediated protection from IL-3 withdrawal, but did not affect AKT activation (Fig. 1).

**Fig 1. Dicoumarol inhibits IGF-I-mediated cell survival and JNK activation.**

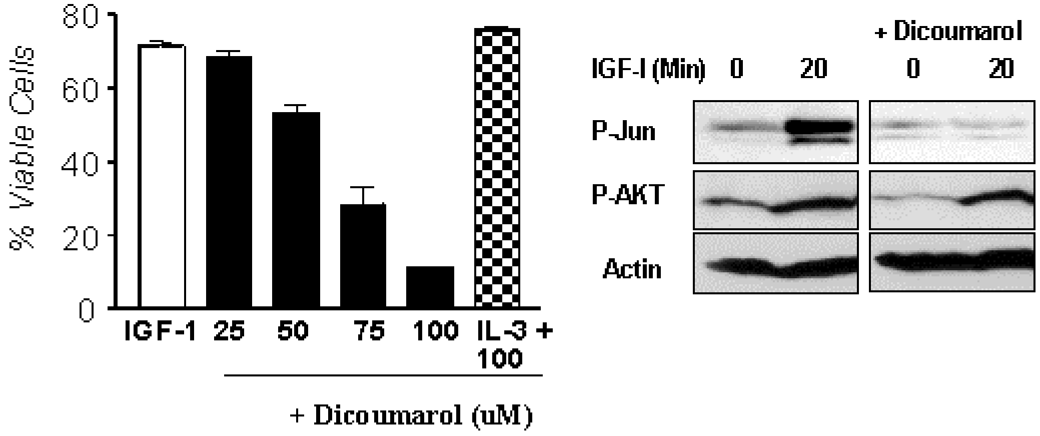

**DISCUSSION.** We found that JNK and c-Jun are phosphorylated by IGF-I stimulation of activated T cells, and as shown in (Fig. 2) IGF-I protects activated T cells from Fas-induced apoptosis. However, this protection is completely suppressed by dicoumarol.

**Fig. 2**

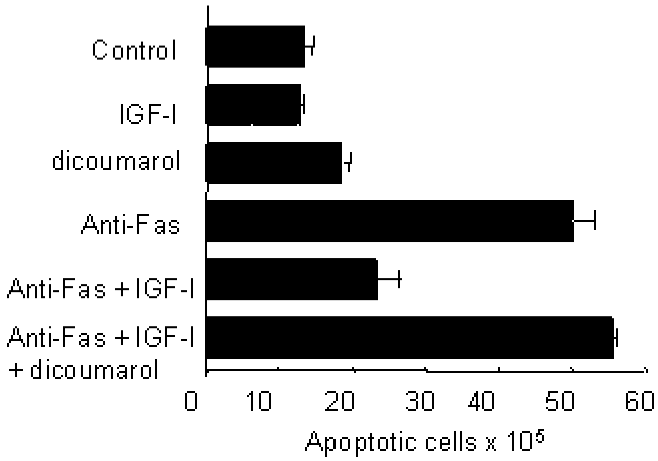

We also assesed the status of the p38 kinase in FL5.12 cells and found that although p38 was constitutively phosphorylated in these cells the p38 inhibitor SB203580 could suppress IGF-I-mediated protection from IL-3 by approximately 50%. Altogether, these data demonstrate that transient activation of Jun kinases by IGF-I occurs in a PI-3 kinase independent manner, and suggests that activated SAPKs are required for IGF-I survival signalling.

**REFERENCES.**

1 O'Connor, R., Kauffmann-Zeh, A., Liu, Y., Lehar, S., Evan, G.I., Baserga, R., and Blattler, W.A. (1997) Mol. Cell. Biol. 17(1), 427-435

2. Cross, J.V., Deak, J.C., Rich, E.A., Qian, Y., Lewis, M., Parrott, L.A., Mochida, K., Gustafson, D., Vande Pol, S., and Templeton, D.J. (1999) J. Biol. Chem. 274(44), 31150-31154

3. Dong, C., Yang, D.D., Tournier, C., Whitmarsh, A.J., Xu, J., Davis, R.J., and Flavell, R.A. (2000) Nature 405(6782), 91-94

4. Walsh, P.T. and O'Connor, R. (2000) Eur. J. Immunol. 30(4), 1010-1018
